# Optimizing Pandemic Preparedness and Response Through Health Information Systems: Lessons Learned From Ebola to COVID-19

**DOI:** 10.1017/dmp.2020.361

**Published:** 2020-10-02

**Authors:** Arush Lal, Henry C. Ashworth, Sara Dada, Laura Hoemeke, Ernest Tambo

**Affiliations:** Women in Global Health, Washington, District of Columbia; Department of Health Policy, London School of Economics and Political Science, London, UK; Harvard Medical School, Boston, Massachusetts; Vayu Global Health Foundation, Boston, Massachusetts; University of North Carolina, Gillings School of Global Public Health, Chapel Hill, North Carolina; Africa Disease Intelligence, Surveillance and Communication; Higher Institute of Health Sciences, University des Montagnes, Cameroon

**Keywords:** COVID-19, pandemic, Ebola, health information systems, health systems strengthening, outbreak response, preparedness

## Abstract

Strengthening health systems and maintaining essential service delivery during health emergencies response is critical for early detection and diagnosis, prompt treatment, and effective control of pandemics, including the novel coronavirus disease 2019 (COVID-19). Health information systems (HIS) developed during recent Ebola outbreaks in West Africa and the Democratic Republic of the Congo (DRC) provided opportunities to collect, analyze, and distribute data to inform both day-to-day and long-term policy decisions on outbreak preparedness. As COVID-19 continues to sweep across the globe, HIS and related technological advancements remain vital for effective and sustained data sharing, contact tracing, mapping and monitoring, community risk sensitization and engagement, preventive education, and timely preparedness and response activities. In reviewing literature of how HIS could have further supported mitigation of these Ebola outbreaks and the ongoing COVID-19 pandemic, 3 key areas were identified: governance and coordination, health systems infrastructure and resources, and community engagement. In this concept study, we outline scalable HIS lessons from recent Ebola outbreaks and early COVID-19 responses along these 3 domains, synthesizing recommendations to offer clear, evidence-based approaches on how to leverage HIS to strengthen the current pandemic response and foster community health systems resilience moving forward.

Health information systems (HIS) are systems focused on health-related “data collection, distribution, and use that provide information at regular intervals.”^1^ HIS are diverse and can include surveillance systems, electronic and mobile health records, health databases, and qualitative and quantitative epidemiological data.^2^ They also collect a wide array of health information to guide decisions at all levels of a health system, from daily management to policy decision-making to implementation. In noncrisis settings, effective HIS can be a critical resource in improving health system function and resilience to public health threats.^1^ In crisis settings, routine and emergency HIS provide real-time, vital information to guide proactive and rapid response activities.^3,4^ Despite the value of robust HIS in responding to pandemics, many countries face challenges in coordinating and expanding HIS, sharing data and information, addressing public mistrust and misinformation, and preventing negative health outcomes during and after outbreaks.^1,3^

Effective HIS implementation could have improved response to the 2014-2016 West Africa and 2018-2020 Democratic Republic of the Congo (DRC) Ebola outbreaks.^4,5^ Three major gaps emerged as opportunities for optimizing HIS: governance and coordination, health systems infrastructure and resources, and community engagement and risk communication. Governance in public health emergencies refers to the political processes and mechanisms that steer response and resilience efforts.^6^ Health systems infrastructure focuses on providing the foundation and resources for planning, delivering, evaluating, and improving public health.^7^ Community engagement includes initiatives that ensure individuals and communities are central in public health priorities and action plans.^8^ As the world responds to the novel coronavirus disease 2019 (COVID-19), recent analysis suggests that strengths and weaknesses in HIS have re-emerged along these 3 domains, offering timely insight on how to scale up evidence-based and efficient HIS to contain the ongoing pandemic.

These emerging themes portray a cyclical relationship; strengthening HIS may lead to more resilient health systems with community-based approaches to emergency preparedness and response, while strengthened health systems provide the foundation for robust and sustainable HIS. Noting the lack of documentation on the implications and added value of scaling up HIS and related digital innovation during public health emergencies, a critical analysis synthesizing concepts along these domains is needed to prevent and mitigate COVID-19 and other emerging public health threats worldwide.

This piece uses examples from Ebola and COVID-19 because of their similarities as fast-moving infectious diseases that have been designated as Public Health Emergencies of International Concern (PHEIC), their high impact on morbidity and mortality, and their disruption of routine health services and economic activity at a global scale. This concept study assesses lessons learned from HIS deployment during the 2014-2016 West Africa and 2018-2020 DRC Ebola outbreaks and recent insights from the COVID-19 pandemic, synthesizing recommendations to optimize ongoing and future preparedness and response actions across 3 overarching domains: governance and coordination, health systems infrastructure and resources, and community engagement.

## GOVERNANCE AND COORDINATION

### Ebola

Different government priorities and agendas are reflected in HIS implementation and have varying implications for how preparedness and response interventions are developed and implemented at all levels of the health system. For example, an agenda centered on global health security has different implications for program priorities and resource mobilization than an agenda focused on universal health coverage—both played an important role in influencing the governance of health systems in Ebola-affected countries.

Governance in West Africa during the Ebola outbreak was weakened not only by the governments’ inadequate coordination and capacity to prevent, detect, and respond to the emergency, but also by the public’s deteriorated trust in government intervention.^9^ Meanwhile, the DRC, an active conflict zone with a complex network of informal politics, offered unique challenges and dangers that impacted HIS implementation.^10^

Robust governance and coordinated responses require cohesive health systems with up-to-date information to enable proactive decision-making, rapid resource mobilization, and effective risk communication strategies; the lack of comprehensive HIS can further fragment these priorities.^1^ For example, during Ebola outbreaks in West Africa, the governments of Liberia, Guinea, and Sierra Leone were slow to initiate necessary emergency response and consistent communication messaging to contain the outbreak.^11^ This was partly due to the lack of existing information systems in place to understand the extent of the outbreak over an expansive geographic area.^12^ Politicization of priorities, complicated frontline actions, and an increasingly complex network of international actors made it difficult to transparently share much-needed data and information on outbreak consequences, weakening rapid response and global solidarity.

For HIS to function effectively, governing bodies and decision-makers need to address barriers to transparent data collection, analysis, and sharing. Notably, the governments of Guinea and Sierra Leone drew criticism for withholding vital information from Médecins Sans Frontières, which was also supporting the Ebola response.^13^ This prevented stakeholders from coordinating health system strategies. Furthermore, the Ebola surveillance system in Sierra Leone was kept distinct from the country’s Integrated Disease Surveillance and Response (IDSR) framework, partially due to administrative and operational barriers such as contested ownership and governance over data.^14,15^ Effective surveillance systems and proactive community contact tracing require robust, coordinated oversight and capability to share data with diverse stakeholders. Furthermore, integrated national roll out of the District Health Information Software 2 (DHIS2)—a free, open source, and locally customizable health management data platform used by over 50 countries—could provide real-time data that governments can leverage to better inform aligned outbreak responses.^1,16^ Promoting efficient and useful HIS solutions, including incorporating epidemic information systems into existing local DHIS2 and IDSR systems rather than running them in parallel, could avert future failures in policy coordination. To improve governance during public health crises, ministries of health should take ownership of and integrate routine and emergency HIS through cohesive governance of data sharing mechanisms and digital risk communication strategies.^14^

### COVID-19

The COVID-19 pandemic has exposed how the chronic lack of HIS within and among nations continues to exacerbate the lack of cohesive national and global health governance. Furthermore, little has been documented on how earlier successes have been deployed and leveraged globally in COVID-19 mitigation and response tactics.

For example, the West Africa 2014-2016 Ebola epidemic showed how weaknesses in governance and data sharing mechanisms hindered coordinated response efforts.^17^ As robust data are the foundation for effective HIS, the World Health Organization (WHO), alongside leaders from low- and middle-income countries (LMICs) and leading scientific journals, developed protocols to streamline the release of data from clinical trials during public health emergencies.^17^ These policy proposals helped lead to the early public release of the COVID-19 genome sequence and the polymerase chain reaction assay protocols, as well as enabled early detection of cases in the COVID-19 pandemic.^18^ This highlights accessible and transparent clinical trial data as an important component of HIS during health crises. However, current mechanisms do not similarly obligate the dissemination of information from observational studies, risk analysis and disease monitoring programs, and outbreak surveillance, thus hindering effective pandemic preparedness and response coordination.^19^

Efficient dissemination of clinical trial results is important to characterize specific disease treatment protocols, but does not fully inform governments on how they should respond to rapidly changing transmission dynamics, asymptomatic reservoirs, and other COVID-19 epidemiological data.^18,20^ As demonstrated during Ebola outbreaks, timely information from contact tracing, disease monitoring, and surveillance are vital to ensure governments can generate up-to-date data and evidence-based policies to spearhead coordinated public health emergency responses.^21^ Consequently, data from observational studies, efficacy of mitigation strategies, and transmission statistics are critical for effective national and international resource mobilization, timely allocation, and technical assistance.^20^ This requires promoting open access distribution and data-sharing mechanisms as an important foundation to improve HIS governance and interoperability before, during, and after health emergencies—building on lessons learned after Guinea and Sierra Leone withheld this vital information during Ebola.^18^

Another important factor that has impaired unified pandemic response policies within several countries and internationally has been the lack of data from cohesive HIS to drive systematic screening. For example, the United States did not begin screening until January 17, 2020, at which point they initially focused only on travelers from China at select airports, even though COVID-19 had already spread to multiple other nations.^22^ This absence of timely information and data sharing leads to weak national and international coordination, affecting logistics in triage and testing, quarantine, and treatment options, as similarly demonstrated by Sierra Leone’s struggle to integrate routine and emergency HIS during Ebola.

One critical HIS solution to streamline screening and build local and national government capacity for informed decision-making is the implementation of a national electronic medical records (EMR) system. Previous studies on national EMRs have demonstrated their ability to enhance screening, reduce medical errors, improve treatment of acute and chronic diseases, and save up to billions of dollars in health-care costs annually.^23,24^ National EMRs can be a constant data stream for HIS during outbreaks, providing detailed information at the individual and population levels to identify cases, track transmission, and support unified government priorities. The success of integrated EMRs has been demonstrated by Taiwan’s response to COVID-19. By leveraging robust information communications infrastructure, national EMRs, and transparent data sharing practices developed following the SARS outbreak, Taiwan has used real-time analytics to stratify risk, screen potential cases, quarantine positive individuals, and treat patients.^25^ Despite significant inbound travel from the epicenter of the COVID-19 outbreak in mainland China, Taiwan continued to have fewer cases than many neighboring countries, with fewer than 450 confirmed cases as of May 5, 2020.^25,26^

The COVID-19 pandemic exposes weaknesses in governing bodies to coordinate responses across diverse contexts and in conjunction with varying global commitments, such as the WHO International Health Regulations (2005) and UN Sustainable Development Goals. While multiple factors play a role, integrated data and information sharing systems are essential in outbreaks like Ebola and COVID-19. Future commitments should include (1) bolstering international cooperation and global solidarity for openly publishing a wide variety of research during health emergencies, and (2) strengthening contextual HIS within countries, including centralized EMRs and interoperable digital platforms that integrate routine and active HIS at all stages of an epidemic.

## HEALTH SYSTEMS INFRASTRUCTURE & RESOURCES

### Ebola

HIS have been leveraged to broadly strengthen health systems and enable the monitoring of ongoing health service utilization during outbreaks.^15^ To facilitate health-care infrastructure capacity-building, HIS could help countries equitably optimize available local and international resources. In the West Africa Ebola outbreak, challenges in health systems infrastructure included scarcity of both physical and personnel resources. Guinea, Liberia, and Sierra Leone have among the fewest physicians per capita worldwide.^27^ Meanwhile, the shortage of personal protective equipment (PPE) was further exacerbated by a lack of procedural systems and accountability mechanisms in place for safe use and removal of PPE.^28^ These scarcities limited the capacity of health systems to mitigate and respond to case management needs in both Ebola treatment centers (ETCs) and other health facilities. Often, assistance was diverted from routine health services, leading to inaccessible care and higher mortality from other diseases (ie, malaria and HIV) than from the outbreak itself.^29^

In West Africa, information about stockpiles and available resources was also hindered by poor telecommunication (especially in rural areas) and a lack of information technology (IT) equipment and expertise, which is vital to capture HIS data during public health emergencies.^30^ Following the West Africa outbreak, partnerships were developed to strengthen digital health infrastructure to support health emergency preparedness.^31^ When the first Ebola outbreak occurred in the DRC in May 2018, the Ministry of Health “called on digital systems” as a key tool in response efforts.^32^ This digital infrastructure would enable HIS to rapidly share and connect up-to-date data, therefore providing greater insight on day-to-day changes at the facility level during outbreaks.

Building up IT and digital resources strengthens surveillance and response systems, which provides early information on case numbers and contact tracing. A study in 2017 evaluating DRC’s Technical Guide for IDSR found that developing an adequate surveillance system was limited by the capacity of the health system itself, including low levels of training and inadequate supporting resources.^5^ Furthermore, at various points throughout the outbreak in Sierra Leone, some hospitals were overrun and had to turn away suspected cases while others were only half-full.^33^ Here, a coordinated HIS could have allowed for more efficient resource and patient allocation across clinics, improving overall care by reducing strain on health facilities. Insight provided by HIS, thus, can be used to inform decisions about physical resource allocation. For example, an integrated HIS in West Africa to provide timely updates on disease hotspots and health facilities could have been leveraged to equitably allocate the limited PPE and avoid stockpiling or supply chain disruptions.^4,34^

These experiences in West Africa highlighted the critical role of IT infrastructure in HIS for outbreak preparedness and response. Therefore, it was promising to see growing use of digital technology to scale up HIS in the DRC 2 years later to guide decision-making where public health infrastructure may be inadequate to meet patient needs.^31^ However, there is still an opportunity to better develop HIS and related IT to maximize resource allocation and quality health service delivery in resource-limited settings.

### COVID-19

In the current COVID-19 pandemic, the scarcity of PPE and emergency supplies has been an issue for even the most well-financed countries and health facilities.^35,36^ Healthcare workers globally have been working without adequate protective equipment and have been forced to decide who should live and who should die through allocation of limited ventilators.^37^ Without information on access and delivery to guide effective redistribution, the lack of these vital resources leads to higher rates of disease transmission and increased mortality, especially in underserved communities—just as they did in West Africa and the DRC during Ebola outbreaks.^36^ Furthermore, mandating increased production of required equipment may only be possible in resource-rich contexts and may still be inadequate to fulfill demands in high-burden areas.^37^ However, strong HIS are essential for the equitable allocation of critical resources in pandemics such as COVID-19, as supply chain requirements vary significantly across locations.

HIS can redirect the allocation of resources precisely to where they are most needed across regions and over time, so that total demand is met within the constraints of limited resources available. Taiwan successfully demonstrated this by leveraging its integrated HIS to analyze multiple national datasets for transparent distribution of PPE to citizens in locations of greatest need.^25^

While nations should incorporate HIS to survey and track internal stockpiles and allocate supplies based on current and predictive needs, the global context must also be considered. Though the majority of COVID-19 cases were initially confirmed in HICs, transmission of the virus exponentially increased in LMICs.^38^ As Ebola outbreaks earlier demonstrated, many LMICs do not have the ability to scale up supply of PPE, and often have inadequate supporting infrastructure to effectively implement nonpharmaceutical interventions, such as physical distancing.^37,38^ Current trends suggest that LMICs, including Sudan, Nigeria, and Kenya are at heightened risk of being devastated by COVID-19 due to dense populations, local conflicts, and fragile infrastructure.^39^ Without the ability to efficiently identify and reallocate excess resources from countries that have reached epidemic control to the most vulnerable regions with unmet demand, COVID-19 is likely to disproportionately impact these vulnerable communities.^39,40^ Effective HIS can address this gap by identifying areas with real-time shortages in supplies and personnel, just as they did toward the end of the DRC Ebola outbreak. This is even more vital in LMICs that cannot rely on other mechanisms to feasibly “flatten the curve.”

Timely support and technical assistance from multilateral organizations, such as WHO and UNICEF, could provide resource development and joint expertise in improving health systems governance and programming through strengthened domestic and global HIS implementation.^41^ Developing HIS within nations could increase global commitments to quality data collection and analysis for evidence-based decision-making. Therefore, global solidarity and investments should be directed toward scaling up HIS and related digital technologies, including real-time data on existing country resources, shifts in geographic hotspots, and infrastructure gaps to curb the spread of COVID-19.

## COMMUNITY ENGAGEMENT

### Ebola

Public health emergency interventions often target human behavior change; such measures require reliable information brokered through effective risk communication and community trust. During the recent Ebola outbreaks, fear and uncertainty of the disease appeared to spread as fervently as the virus itself. The mounting public panic paved the path for widespread misinformation and rumors, seeded by convoluted messaging and a chronic lack of trust in domestic governments and foreign intervention.^33,42^ This delayed control measures, as sociopolitical factors were not integrated into HIS to guide pragmatic response strategies at the local level. For example, in West Africa, rumors that ETCs were dangerous negatively influenced health-seeking behavior.^43,44^ In the DRC, 2 decades of ongoing conflict added layers of community mistrust of government officials and humanitarian aid organizations.^45^ Low levels of trust hampered outbreak control programs and fueled attacks on health-care workers and ETCs.^46^ Community engagement and open dialogue involving local leaders gradually restored trust and eventually provided critical pathways for information to support HIS ownership and guide future outbreak preparedness and response.^33^

In West Africa, the transition from ineffective “sensitization” messages solely aimed at correcting misinformation to bottom-up communication campaigns involving local communities was a turning point in the epidemic response, as it empowered community-based management through informal information systems.^47-49^ In Sierra Leone, community event-based surveillance (CEBS) systems were facilitated by information about social and ecological contexts from local community volunteers to detect, isolate, and treat potential cases to prevent further transmission.^50^ To be effective, CEBS required local buy-in and relied on relationships among volunteers, communities, and external stakeholders to supplement HIS that tracked clinical and epidemiological data but lacked social context.^51^ Integrated HIS could thus provide guidance to collect and monitor more context-specific information.

While the importance of community engagement has been emphasized in outbreak response, established strategies for sustainable integration of relevant indicators into HIS have not been clearly determined.^52^ Lessons from West Africa pointed to the significant role of chiefs and local leaders, but also emphasized informal structures of power, suggesting the need for community participation in HIS decision-making and implementation.^53^ Further strategies included recruiting local staff for social mobilization, working with religious leaders, engaging local radio and journalists, and hosting community meetings—all possible sources for relevant information in HIS.^54,55^

Notably, the International Federation of Red Cross and Red Crescent Societies (IFRC) published a guide outlining a 5-step process for community engagement to collect data for effective decision-making that was implemented in the DRC outbreak, with a network of volunteers and an extensive database to collect and track rumors and community perceptions as the outbreak transpired.^56^ This demonstrates how HIS, when leveraged effectively, can be used to incorporate data related to contact-tracing networks as well as relevant experiences, perceptions, and rumors that may hinder response efforts. This work has been conducted mostly by anthropologists, but there is a need to conduct analyses that can be systematically replicated, validated, and compared.^49^ A robust HIS can offer the possibility of collecting and systemizing salient social data, and allow communities to be actively involved in informing real-time insights that improve preparedness and response efforts.

### COVID-19

While the role of HIS in bolstering community resilience and engagement to strengthen outbreak response has been documented following the West Africa and DRC Ebola epidemics, similar mistakes are still being made in addressing the COVID-19 pandemic.^53^

One of the biggest sources of mistrust has been the circulation of false and misleading information about COVID-19 prevention and treatment options.^57^ For example, a lack of effective COVID-19 communication in Iran’s response programming continues to fuel mistrust and inhibit adequate emergency measures, mirroring poor risk communication practices from prior Ebola outbreaks.^58^ While credible authorities, such as the WHO and the Centers for Disease Control and Prevention (CDC), have routinely provided up-to-date information, their social media posts have only reached several hundred thousand social media engagements, such as liking or sharing a Tweet or Web page. This level of credible social media engagement is dwarfed by the over 52 million engagements garnered by hoax and conspiracy theory websites by March 2020, highlighting the challenge of curtailing “viral” fake and misleading news that spreads quickly among the general public.^57^ Social media posts with disinformation have been increasing, often with racist and xenophobic remarks particularly aimed toward people of Chinese origin living in other countries, as exemplified by the hashtag “#ChineseDontCometoJapan” trending on Twitter.^59^ The lack of effective surveillance, tracking, and accountability of these xenophobic statements by current systems means governments struggle to forecast their negative effects. This has further isolated key populations, stigmatized reporting of symptoms, and fueled prejudices that may harm communities well beyond the current crisis.^60^ The WHO refers to this as the “Coronavirus Infodemic” and has noted that tackling misinformation is just as important as fighting the pandemic itself.^61^ Similar to challenges faced in communicating preventive practices at the start of the West Africa Ebola outbreak, it is clear that initial strategies for disseminating accurate information and risk communication for COVID-19 are not working, and the role of community trust is, thus, more critical than ever.

HIS have been used to generate valid scientific data, but are not being used to identify at-risk populations to effectively engage. Strategies the WHO has implemented in the COVID-19 pandemic include directly engaging with social media platforms and ensuring partner websites have banners linked with accurate information.^61^ HIS could aid in addressing the current COVID-19 infodemic and future health crises through 2 additional mechanisms. First, integrated HIS could help identify vulnerable populations at greater risk of COVID-19 exposure, detailing community engagement methods supported by data collected at the community level. Vietnam, similar to the DRC during Ebola, has ensured a strong focus on engaging communities through media directed by medical experts, which has proven extremely effective in reducing transmission of COVID-19.^62^ However, more comprehensive HIS could be used to develop individualized programs that meet the needs of specific communities with targeted interventions, including tailored information campaigns, socioeconomic support, and access to health-care resources. Second, just as contact tracing identifies people potentially exposed to COVID-19, HIS could include information about the source and spread of misinformation and create counter-campaigns with accurate information, drawing from IFRC’s 5-step process for community engagement in the DRC Ebola outbreak. Powerful analytics such as artificial intelligence (AI) and big data technologies could be leveraged to ensure that these procedures are conducted effectively while also protecting privacy.^63^ Robust HIS could, therefore, refine risk communication that builds trust as well as informs new population-specific community engagement methods.

## NEXT STEPS

Strengthening contextual HIS are critical to inform outbreak response, but also generate evidence to build resilient health systems. Exploring and applying HIS lessons from previous Ebola outbreaks provides an opportunity to improve pandemic preparedness, mitigation, and response strategies for other emerging diseases. These experiences are of particular value to the ongoing COVID-19 pandemic. The uncertainties fueled by rapid disease progression and a lack of effective information systems across local, national, and global levels exposes evolving gaps in global HIS that should be addressed to curb transmission and mitigate downstream health and socioeconomic impacts.

### Governance and Coordination

Strengthening local and regional HIS governance and coordination provides vital data that allows governments to make real-time policy decisions, allocate resources, and inform preparedness plans. Priorities should be aligned across global health agendas to reduce fragmentation of policies and programs that constrain HIS during health emergencies. At a national level, recent Ebola outbreaks have demonstrated the urgency for integrated routine and emergency surveillance systems to avoid redundant or parallel systems, and COVID-19 has underscored the need for improved data streamlining and data sharing at all levels of governance.^39^ At an international level, reforms from the Ebola outbreaks have led to improvements in sharing of clinical trial data.^18^ We recommend further reforms so that other sources of vital HIS data, including observational studies and disease monitoring and surveillance programs are openly shared. This is critical to ensure all countries have the necessary information to implement rapid protocols and strategic plans for potential pandemics such as COVID-19.^41^

### Health Systems Infrastructure & Resources

Building health systems infrastructure requires scaling up HIS implementation and integration in primary health-care programs to help efficiently direct key resources like PPE and health workers. The West Africa and DRC Ebola outbreaks demonstrated the need for stronger HIS and enhanced IT capacity to manage limited resources. In contrast, Taiwan’s COVID-19 pandemic response that leverages an integrated, national EMR has shown how robust HIS can be a vital tool to support contact tracing, surveillance, and PPE distribution in the context of scarce medical supplies.^44^ While the measures implemented in Taiwan may not be achievable everywhere, even small investments in health systems infrastructure may yield massive health gains, before, during, and after a crisis.^43^ We recommend that as countries strengthen their HIS, they consider investments in IT and national EMRs as mechanisms for responsive, accountable, and equitable resource allocation. Centralized databases of HIS may also enable novel analysis with the help of AI and other innovative technologies.

### Community Engagement

Promoting community engagement and resilience is crucial in responding to emerging disease outbreaks and other health emergencies. The Ebola epidemics and ongoing COVID-19 pandemic demonstrate how local context affects adequate response, thus emphasizing community participation as a critical component at all stages of emergency preparedness. HIS provide a platform to employ and adapt community empowerment and engagement. First, communities can guide consideration, prioritization, and decision-making for which data are gathered and analyzed. This would improve accuracy and build local trust so that HIS could be used as they were in Taiwan to identify, track, and treat high-risk populations with targeted approaches.^25^ Second, addressing rumors and misinformation is vital in stopping transmission and improving cooperation for better health outcomes.^53^ Therefore, HIS should be developed and implemented to address the “infodemic” by helping identify sources of misinformation and generating targeted evidence with relevant information for specific communities.

## CONCLUSION

The failures of health systems in the previous Ebola outbreaks and a globally catastrophic pandemic like COVID-19 are often discussed, but may overlook the critical role of information systems in optimizing decision-making and health emergencies response. Robust HIS that are adequately financed and developed before a future outbreak can cyclically strengthen health systems and pandemic preparedness and response capacities. High-performing and effectively leveraged HIS are catalytic in informing coordinated global health governance and ensuring timely, transparent data sharing. They strengthen health infrastructure by reducing fragmentation and costs while streamlining equitable resource allocation. They offer new ways to foster community engagement, combat misinformation, and cultivate trust.

As COVID-19 has shown, major pandemics can drive even the most stable economies into fragile conditions while forcing already-fragile contexts into more vulnerable states. Investments in HIS can prevent this cascading deterioration of global economies and security by offering timely insights as a backstop to enable national governments to quickly prevent, detect, and respond to public health threats, fostering more robust response and recovery interventions and building a resilient post-COVID-19 world.
